# Dermatology undergraduate skin cancer training: a disconnect between recommendations, clinical exposure and competence

**DOI:** 10.1186/1472-6920-12-27

**Published:** 2012-07-09

**Authors:** R Benjamin Aldridge, Susanne S Maxwell, Jonathan L Rees

**Affiliations:** 1 Department of Dermatology, University of Edinburgh, Level 1 Lauriston Building, Lauriston Place, Edinburgh, EH3 9HA, UK

## Abstract

**Background:**

Skin cancers are the most common malignancies in Caucasian populations. Non-specialists are responsible for the initial assessment of skin lesions and are required to act as the gatekeepers to dermatological cancer services in many healthcare systems. The majority of such physicians receive very limited formal undergraduate or postgraduate dermatology training. The British Association of Dermatologists (BAD) has produced guidelines that list the lesions that students should be able to diagnose on graduation and the majority of UK medical schools’ operate curricula in keeping with these. There is, however, virtually no evidence as to whether these competencies are being achieved. We set out to determine students’ competence at skin lesion diagnosis and to quantify their clinical exposure to examples of such lesions during their dermatology attachment.

**Methods:**

Three linked studies were undertaken. In the first, students’ competence was tested by randomized slideshows of images containing the 16 lesions recommended in the UK guidelines. Students’ accuracy was tested at the beginning (Day 1) and end (Day 10) of their clinical placement, with a random sample of students retested 12 months later. Secondly, students’ exposure to these lesions was recorded during their attachments. Finally a survey of the additional dermatological resources used by the students was undertaken.

**Results:**

Study 1: Students’ diagnostic accuracy increased from 11% on Day 1 to 33% on Day 10 (effect size +2.72). After 12 months half of this effect had disappeared and the students accuracy had dropped to 24%. Study 2: Students’ exposure to the recommended lesions was poor with 82% not even witnessing a single example of each of the 3 major skin cancers. Despite these measurements, only a minority of students reported that they were not confident at diagnosing skin tumours. Study 3: The majority of students use additional resources to supplement their learning.

**Conclusions:**

In the light of what we know about learning in dermatology, our data would suggest, that the current (traditional) undergraduate attachment is inadequate to meet the UK recommendations for graduate competence. As well as critically examining the basis for these recommendations, we need more empirical data on student performance and exposure, in order to improve teaching and learning.

## Background

Cutaneous malignancies account for over a quarter of all new ‘cancer’ diagnoses in the UK [1]. Diagnosing skin cancer is largely a perceptual skill, relying little on formal or explicit rules, but rather on prior exposure and feedback either in a training environment or in the clinic. Following Norman’s terminology, the skills involved are largely thought to be those of non-analytical pattern recognition (NAPR), and this core skill can be viewed as being able to attach semantics to images or percepts [2-10].

In the UK general practitioners are responsible for the initial assessment of skin lesions and act as the gatekeepers to dermatological cancer services. For most of these medical practitioners their only formal clinical dermatology teaching is as an undergraduate. Despite the fact that cutaneous disease has been shown to be the most common reason for primary care consultations (at 24% of physicians’ new patient workload) [11], medical school training in dermatology is relatively limited contributing to only ≈ 1% of undergraduate teaching time [12].

Whilst historically each UK University has set its own curricula, in the light of recommendations from the General Medical Council (GMC), in 2006 the British Association of Dermatologists (BAD) produced the recommendations “Dermatology in the Undergraduate Medical Curriculum”, outlining what ought to be expected of students upon graduation [13]. These guidelines were based on the results of a modified Delphi study that had been conducted with a multi-disciplinary panel of 66 individuals involved in delivering undergraduate education [14]. Under the “skin cancer” section the guidelines state that it is very important that graduates should be able to recognize the three main skin tumours (basal cell carcinoma (BCC), squamous cell carcinoma (SCC) and melanoma) and that it is fairly important that they can recognize another 13 common skin lesions — diagnosing implicitly means distinguishing the rarer malignant lesions from the more frequent benign ones. A 2009 audit of UK medical schools’ curricula concluded that these “skin cancer” learning outcomes were present in the syllabuses of most universities [15]. Of course because a topic is present in a curriculum does not mean that a particular student competence is actually achieved.

Given the paucity of research work in this area — even of observational studies — we wished to collect evidence of our students’ competencies in this clinical domain and relate them to clinical exposure and student confidence. How much exposure to skin cancers do students actually gain as an undergraduate and what do the prescribed competencies mean in practice? We show that whilst current teaching has a measurable effect on improving students’ diagnostic accuracy, half of that effect is transient, and furthermore students are not exposed to enough examples to realistically meet the suggested UK graduate competencies.

## Methods

The University of Edinburgh’s undergraduate dermatology teaching program is similar to the majority of other UK Medical Schools [12]; consisting of an introductory series of lectures, followed by clinical exposure in outpatients. In Edinburgh there are 9 lectures and 10 half-day clinical sessions over a two-week attachment undertaken in the student’s penultimate clinical year. The clinical sessions incorporate 8 small group demonstration clinics where the instructor has no clinical responsibility and where patients are obtained from up to 10 adjacent NHS feeder clinics, a “one-on-one” NHS clinic with a consultant, and a skin surgery session. The whole attachment is undertaken in the regional teaching hospital, with a departmental throughput of in excess of 25,000 new patients per annum.

Study 1: Student competence at skin tumour diagnosis

All 77 students undertaking their dermatology attachment between July and September 2009 were enrolled. Fifty students (65%) were female. Over the ten-week study period, 5 cohorts of between 14–17 students attended. The students’ diagnostic accuracy was examined using a digital slideshow, with the students writing diagnoses on a custom designed answer sheet. We were “generous” in what we accepted as correct answers, allowing spelling mistakes, incomplete terminology, abbreviations and lay terms. Assessment was undertaken prior to seeing any patients on the first morning of the attachment (Day 1 test) and again on the final afternoon (Day 10 test). Note that the students are not formally examined at the end of each two-week dermatology block, but undertake a joint exam with other disciplines at a later date. Five separate test batches of images were constructed so that both the Day 1 and Day 10 tests were different for each group of students. Each test batch consisted of 25 digital images randomly selected in a stratified manner to ensure that each test contained an identical spectrum of lesional diagnoses. The 25 lesions, which were presented for each test batch in a different randomized order, included one or more examples of each of the 16 lesions considered important in the UK guidelines. The test images were randomly selected from 687 suitable digital photographs in the Department of Dermatology’s image database. This image library (now standing at over 4000 lesions) has been prospectively collected for research into diagnostic expertise and automated diagnostics. Although the images are not from consecutive patients the library is designed deliberately not to be a typical departmental library of “classic cases” but to represent cases from routine clinical practice. All the images in the library were captured using the same controlled fixed distance photographic setup; Canon (Canon, Japan) EOS 350D 8.1MP cameras, Sigma (SIGMA, Japan) 70 mm f2.8 macro lens and Sigma EM-140 DG Ring Flash at a distance of 50cms. The digital slideshows were conducted in an identical manner using iPhoto on a 15” Apple Macbook Pro (Apple, California) connected to an Epson H285B high definition projector (Epson, Japan), under similar ambient lighting conditions in the Department’s seminar room. The 25 lesions were all presented at the same scale for 20 seconds each. Students were not given feedback after the Day 1 test, but after completing the Day 10 test underwent an addition tutorial during which feedback was provided. 12 months later, in August 2010, 30 of the original students were randomly selected, contacted by email and asked to return to the department. The students were not informed of the reason that they had been selected and asked to return, but they were advised that they would be helping out with a fellow student’s SSC project and would be remunerated for their time at a level of £10. Nineteen students (63%) were able and willing to attend for retesting (12 Month test). The batches of test images used for the 12 Month test were coordinated so that each student re-attending had not been exposed previously to the same digital photographs. The slideshow was conducted in an identical manner as described above for the Day 1 and Day 10 tests.

Study 2: Clinical exposure to skin lesions during the attachment

Over a six-week period the total clinical exposure for each of the 50 students who attended their undergraduate dermatology attachment from July to August 2010 was assessed. Three cohorts of between 16–17 students were monitored. Every clinical visual encounter that the students were exposed to was recorded on a purpose-designed checklist. This checklist contained the same 16 skin lesions considered important in the UK guidelines and was completed by each student for their “one-on-one” teaching and skin surgery sessions, whereas for the group demonstration clinics it was completed by a dedicated clinical observer. If a diagnosis was not on the checklist additional space was available to record text for “other” diagnoses. If more than one lesion was seen on the same patient each lesion counted as a separate clinical encounter. In contrast, if a patient was being followed-up or being used to demonstrate a “dermatological history”, unless there was also a visual example of a skin lesion, these patients did not count as a clinical encounter under the terms of reference of the present study. Medical staff involved in the teaching clinics were not pre-warned that the monitoring process was being undertaken.

Students were asked to complete an anonymous questionnaire on the final afternoon of their attachment (Day 10). The questionnaire asked them to rate their abilities on 7-point Likert scales across the three key skin cancer learning outcomes; their ability to diagnose melanomas, their ability to diagnose basal cell carcinomas and their ability to diagnose squamous cell carcinomas.

Study 3: Learning exposure outside the clinic

To gain a measure of other non-patient visual dermatology exposure, all students completing their dermatology attachment between July and September 2010 (n = 50) and again between March and May 2011 (n = 61) were asked to complete an anonymous questionnaire about the additional resources they had used to supplement their formal teaching. In total 106 (95%) students completed the questionnaire. The purpose-designed questionnaire had tick boxes covering the most popular textbooks and Internet sites and also space to add supplementary free-text if the resource used was not listed. In addition to assessing the types of resources used the questionnaire also asked the students to estimate how long on average they spent using these resources.

### Statistics

Data was tabulated in Excel (Microsoft, California) then exported into R for graphing and statistical analysis [16].

### Ethics

The NHS Lothian ethics committee granted permission for the collection and use of the images, and additional permission for studies of student learning were granted from the University of Edinburgh College of Medicine and Veterinary Medicine students’ ethics committee.

## Results

Study 1: Student competence at skin tumour diagnosis

**Figure 1:**
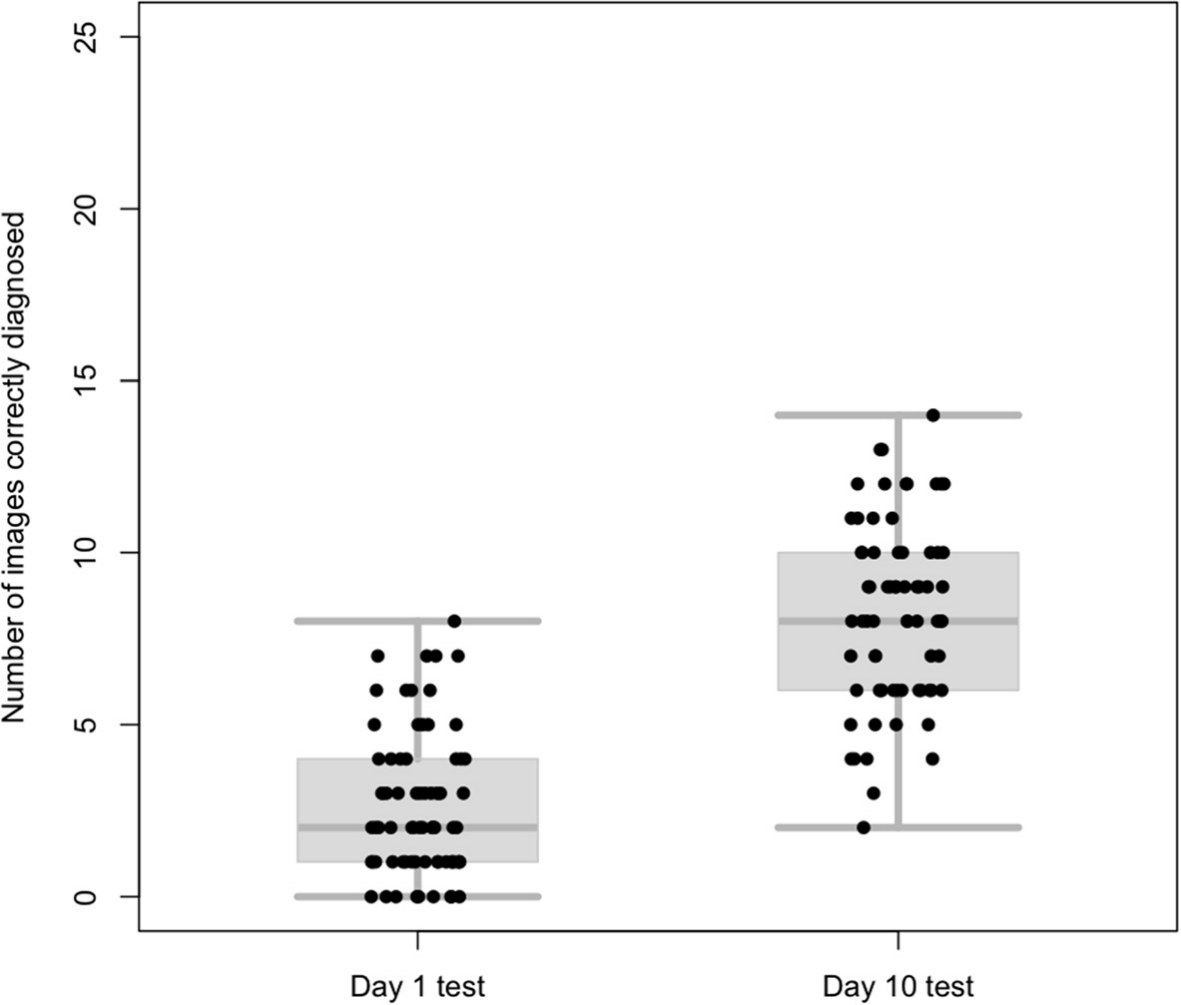
Combined box-plots and scatter plots showing all the test scores for students completing the Day 1 test (n=74, median=2) and the Day 10 test (n=70, median=8).

Of the 77 students enrolled, 74 and 70 completed the Day 1 and Day 10 tests respectively and their scores for the Day 1 and Day 10 tests are shown in Figure 1. In the Day 1 test, out of the 25 test images, the median number of images correctly identified was 2 (Range 0–8), with an overall diagnostic accuracy of 11% (195/1850). In the Day 10 test the median number of images correctly diagnosed was 8 (Range 2–14), with an overall accuracy of 33% (575/1750). Sixty-seven (87%) students completed both the Day 1 and Day 10 tests and the difference between these students’ results on Day 1 and Day 10 was significant (p < 0.0001, Paired Wilcoxon). The overall Effect size (μ1-μ2/σ) of the dermatology attachment on students’ diagnostic accuracy was +2.72 [17]. No differences were observed between the sexes, the cohorts of students or the individual 25 image test batches.

**Figure 2:**
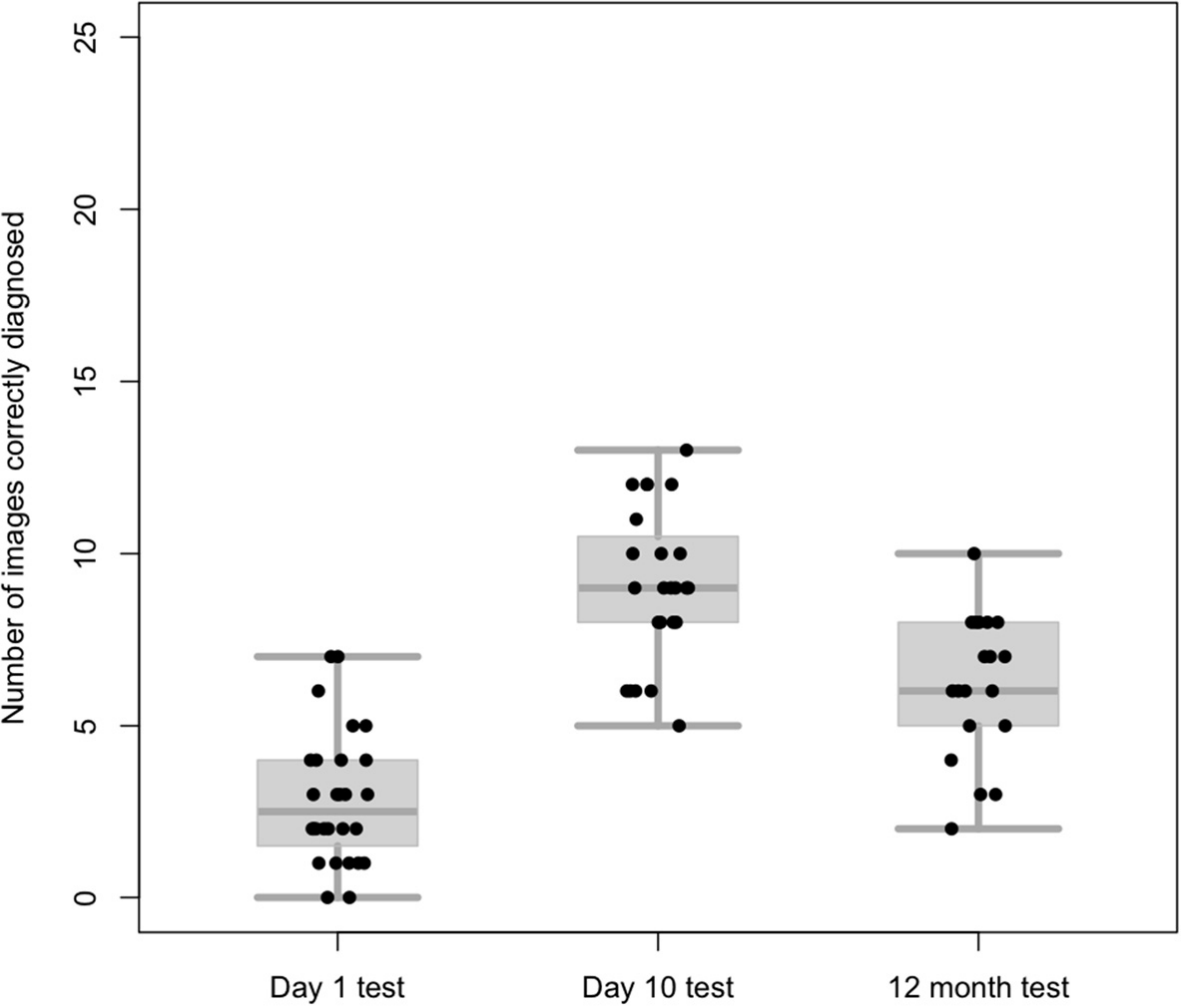
**Combined box-plots and scatter plots showing all the test scores for the 30 students randomly recalled for re-testing after 12 months.** Day 1 test (n=28, median=2.5), Day 10 test (n=24, median=9) and 12 Month test (n=19, median=6).

The scores for the 30 students that were randomly selected for recall after 12 months are shown in Figure 2. In the Day 1 test these students achieved a median score of 2.5 (Range 0–7) with an accuracy of 11% (80/700) and in the Day 10 test a median score of 9 (Range 5–13) with an accuracy of 36% (217/600). After a year their median score was 6 (Range 2–10) with an overall accuracy of 24% (117/475). A Linear mixed-effects model confirmed that there were significant differences between the Day 1 and Day 10 scores, the Day 10 and 12 Month scores and the Day 1 and 12 Month scores (p < 0.001 for each). For this recalled subgroup the initial effect size was +3.1 dropping after a year to +1.59. Inspection of the data suggested that the 19 students who attended for the 12 Month test were representative of the original 77 students enrolled in terms of their sex distribution and original scores on both the Day 1 and Day 10 tests. Similarly, the 11 students that were randomly selected but unable to attend for re-testing also appeared similar in the original diagnostic scores to the 19 students that did attend. Again, in the test at 12 months, no differences were observed between the sexes, the cohorts of students or the individual test batches.

Study 2: Clinical exposure to skin lesions during the attachment

The 50 students saw a median of 23 lesions (7–36) during their dermatology attachments. The three cohorts of students differed significantly in the number of lesions witnessed with medians of 23, 18 and 31 (Kruskal-Wallis P < 0.001). The median number of the 16 important diagnoses witnessed by each student was 8 (Range 5–11) but this did not vary by student cohort. None of the students witnessed an example of all 16 lesions listed in the UK learning outcomes. The overall observation rate, as defined as the number of positive visual encounters that the 50 students witnessed across the 16 recommended lesions, was only 53% (426/800). A full breakdown of the results are in Table 1. The most common lesions witnessed were solar and seborrhoeic keratoses with all the students exposed to at least one example of each. The median number of BCCs seen was 5 with 98% (n = 49) of students witnessing at least one example, for SCCs the median witnessed was 1 and 76% (n = 38) of students saw an example. For melanomas only 38% (n = 19) of students saw an example with the median witnessed being 0. The overwhelming majority of students 82% (n = 41) did not see an example of each of the three major skin cancers (BCC, SCC, melanoma) and only a single student (2%) witnessed two examples of each. The percentage of students witnessing 1, >3 and >5 examples is given for each of the 16 lesions and demonstrates that there was not only a lack of breadth but also of depth to the students’ exposure.

**Table 1:** Table showing the median number of lesions that the students witnessed, split across the 3 cohorts of students for all 16 lesions contained in the UK guidelines

**UK “Recommended lesions”**	**Student cohort 1 (n=16)median exposure**	**Student cohort 2 (n=17) median exposure**	**Student cohort 3 (n=17) median exposure**	**Overall student median exposure n=50 (Range)**	**Percentage of students observing 1 or more example of each lesion**	**Percentage of students observing >3 examples**	**Percentage of students observing >5 examples**
**Individual lesions “Very Important”**							
BCCs	6	2	6	5 (0–11)	98%	70%	42%
SCCs	0	2	1	1 (0–4)	76%	2%	0%
Melanomas	1	0	0	0 (0–2)	38%	0%	0%
**Individual lesions “Important”**							
Viral warts	0	0	0	0 (0–1)	28%	0%	0%
Epidermoid cysts	0	0	0	0 (0–1)	10%	0%	0%
Melanocytic naevi	1	1	5	2 (0–7)	72%	30%	14%
Seborrhoeic keratoses	6	4	8	6 (1–10)	100%	78%	56%
Solar keratoses	3	4	5	4 (1–7)	100%	60%	12%
Bowen’s disease	1	1	2	1 (0–4)	96%	6%	0%
Dermatofibromas	1	1	1	1 (0–3)	80%	0%	0%
Keratoacanthomas	1	2	0	1 (0–2)	64%	0%	0%
Lipomas	0	0	0	0 (0–1)	6%	0%	0%
Pyogenic granulomas	1	0	1	1 (0–2)	54%	0%	0%
Mycosis fungoides	0	1	0	0 (0–2)	28%	0%	0%
Paget’s disease	0	0	0	0 (0–1)	2%	0%	0%
Cutaneous metastases	0	0	0	0 (0–0)	0%	0%	0%
**All 16 lesions**							
Total number of lesional diagnoses seen	9/16	8/16	8/16	8 (5–11)	**426/800 (53%) Overall Observation Rate**	123/800 (15%)	62/800 (8%)
Total number of skin lesions seen	23	18	31	23 (7–36)	NA		

Forty-four students (88%) completed the end of attachment questionnaire. The scores for this student self-assessment questionnaire are presented in Table 2. At the end of their attachment only 34%, 14% and 27% of students described themselves as not confident at identifying melanomas, BCCs and SCCs respectively. Despite the different levels of exposure between the 3 cohorts of students there was no difference between their confidence scores.

**Table 2:** Students’ self-assessment of their own abilities at diagnosing the 3 major skin cancers on completion of the dermatology attachment

**Questions:** How confident are you in your ability to diagnose…	**Mean Likert Score (Range)**	**Median Likert Score**	**Percentage of students “unconfident” (score <4)**
…melanomas?	4.2 (2–6)	4	34% (n=15)
…squamous cell carcinomas?	4.3 (2–7)	4	27% (n=12)
…basal cell carcinomas?	4.8 (2–7)	5	14% (n=6)

44 completed the anonymous questionnaire in which they rated themselves on 7-point Likert scales (1=Extremely unconfident, 2=Unconfident, 3=Reasonably unconfident, 4=Not unconfident, 5=Reasonably confident, 6=Confident, 7=Extremely confident).

Study 3: Learning exposure outside the clinic

Ninety-two percent (97/106) of students used additional textbooks of whom the majority, 58% (56/97), used more than one book. Sixty-five percent (69/106) of students used online resources but only the minority, 43% (30/69), of these students used more than one site. All the resources used by the students are itemised in Table 3. The most popular resource was the New Zealand Dermatological Society’s Website [18]. Students reported spending a median extra 1–2 hours/day studying dermatology during their attachment.

**Table 3:** The supplementary resources used by students (n=106). Answers from an anonymous end of attachment questionnaire

**Internet resources (n = 69)**	
**Website**	**No. of Students (%age)**
DermNet NZ [18]	59 (85%)
University of Edinburgh [19]	25 (36%)
Wikipedia [20]	12 (17%)
BAD [21]	5 (7%)
E-medicine [22]	2 (3%)
PCDS [23]	1
Patient.co.uk [24]	1
**Textbook resources (n = 97)**	
**Textbook**	**No. of Students (%age)**
Davidson’s Principles and Practice of Medicine [25]	57 (59%)
Clinical Dermatology [26]	53 (54%)
Dermatology: an Illustrated Colour Text [27]	31 (32%)
Oxford Handbook of Clinical Specialties [28]	14 (14%)
ABC of Dermatology [29]	9 (9%)
Crash Course [30]	5 (5%)
Physical Signs in Dermatology [31]	4 (4%)
Fitzpatrick’s Dermatology in General Medicine [32]	3 (3%)
Differential Diagnosis in Dermatology [33]	2
Rook’s Textbook of Dermatology [34]	1
Lecture Notes: Dermatology [35]	1

The total number of students using each resource is given along with the relative percentage of students for that type resource (either internet or textbook). The percentages do not add up to 100% as some of the students used multiple resources.

## Discussion

In the first study we showed that students’ diagnostic accuracy for the 16 “important” skin lesions was only 33% after completing their dermatology attachment and that it dropped to 24% a year later. Such absolute measures of diagnostic competence are obviously incomplete and potentially misleading because they are so dependent on the difficulty of the specific tests. However, in the absence of normative data elsewhere in the literature, they provide some sort of benchmark for future work, and other authors are welcome to use our test-sets.

A potential reason for what we take to be the students’ poor accuracy was identified in our second study, where we showed that students’ exposure to the 16 important skin lesions is highly variable but universally limited. Some limitation in clinical exposure is to be expected but, rightly or wrongly, we were shocked by how limited the extent of clinical exposure was. Although there is no systematic data on this issue we suspect that the clinical exposure our students gain is typical of, if not better than that at many other UK centres, and our course duration is actually greater than the UK average [12]. Of note our course scores highly in terms of student feedback compared with other clinical attachments within the University of Edinburgh. In addition, due to the structure of our group demonstration clinics (which allows multiple students to see a single patient) the overall melanoma witnessing rate of 38% was predominately achieved because of a single case -if this one patient had not presented and agreed to be examined at the time of a demonstration clinic the overall melanoma observation rate would have dropped to 18%.

The students’ low exposure demonstrates the major difficulty encountered when teaching students to identify skin cancers and their mimics — a lack of reliable clinical examples. Unlike other specialties where pertinent clinical signs are often longstanding and can be demonstrated repeatedly to different groups of students, suspected skin malignancies are excised in dermatology outpatients as a matter of priority. Therefore if face-to-face patient teaching is to be relied on a constant throughput of new patients is required.

It might be argued that “skin cancer” education is not performed exclusively during students’ dermatology attachments but that additional teaching is happening during their allied clinical attachments. We are sceptical of this and note that our students’ scores, which were lower one year after their dermatology training, followed their 2-month attachment in primary care.

The finding that there appeared a mismatch between objective ability and self-confidence again should not be surprising. Although subjective measures are in widespread use for assessing teaching and learning, there is increasing acknowledgment that students’ (and other health professionals’) self-assessment of their own abilities are often erroneous [36,37]. An additional reason for the students’ overconfidence could also be that the lesions encountered during their teaching attachment were not suitably representative of the variety of presentations that had been randomly selected in our test slide shows (and could be argued are encountered in real-life). Neither the lesions that the students witness in the group demonstration clinics nor those that they are exposed to in their additional study are unselected. In our experience both textbook images and the demonstration clinic lesions are often chosen precisely because they are “classical” examples. Irrespective of the cause, the mismatch in confidence and competence at the end of students’ only formal dermatology training has implications on the role of non-experts as gatekeepers to cutaneous cancer services.

Given the potential importance of undergraduate dermatology education it is surprising that there has been so little investigation of UK medical school dermatology teaching [12,15,38-43]. Whilst additional studies have subjectively shown that both medical students and primary care physicians feel dermatology training could be improved [44-46], there are only a few US studies that have objectively assessed students’ diagnostic acumen after undergraduate dermatology electives [47-49]. These studies are, however, unlikely to be transferable to UK undergraduate students, and are not directly comparable to our study because the difficulty of the questions was not controlled for. Whilst the US studies demonstrated a higher level of improvement in diagnostic competence than we witnessed (71-82% accuracy), there are a number of other potential reasons for this. First, in these studies there could be an element of selection bias; the attachments were not compulsory and the students enrolled had volunteered to undertake dermatology electives, many presumably with the intention of pursuing a dermatology career. Second, the studies used the same images for both the initial and final assessments, which are likely to have artificially raised the final scores. More importantly the test contained not just lesions but also dermatoses and the images used for testing were not randomly selected, instead, the images chosen were often described as “classical” examples. Third, the answers were in multiple choice format rather than free text, which does not correspond to everyday practice. Finally these optional electives were of longer duration than in the UK, being full-time for 2–4 weeks.

We believe the biggest difficulty in interpreting our work is the lack of a coherent and justified framework for the purpose, standards and remit of undergraduate dermatology education. Although the BAD have produced guidelines that have been integrated into UK medical schools’ curricula, there are no specific criteria for any of their recommendations. As a result the current statements, such as “graduates should be able to recognize melanomas”, are as lacking in precision as saying a mathematics graduate should be able to compute. If the guidelines are to be adopted successfully, these criteria need to be objectively defined. This requires that we have to tackle exactly how difficult particular cases are with reference to some common standard.

So how could students’ skin lesion identification be improved? Our own students clearly seek out online resources, and the number of images available online far exceeds that available in the clinic or in textbooks. Online content is, however, very variable in quality and many online images are of poor quality and not infrequently in our experience the diagnosis is questionable, if not wrong. Whilst we know of no experimental work directly comparing learning on real patients versus learning from image databases for skin cancer we strongly suspect that the latter is the way to proceed. Modern imaging techniques already allow for accurate 3D models of skin lesions to be captured simply and efficiently [50]. These models have significant advantages over conventional 2D photography as they allow students to rotate and pan around the images as they would in real-life. A large database of such images could address the main obstacle to effective dermatology teaching by removing our reliance on a constant supply of new example lesions. Furthermore if the images were available online, widespread access could be achieved with relatively low production costs. This would allow standardized exposure across multiple institutions, limiting the intrinsic fluctuations of exposure that occur between students with present teaching arrangements.

## Conclusion

This work suggests that the traditional dermatology teaching attachment is inadequate to meet the current UK guidelines for graduate skin cancer competencies. We note that these guidelines have little objective evidence base to ensure their practicality or validity, and realize that some would argue that it is not reasonable to expect students to be able to diagnose skin cancers to a defined standard. We differ, and furthermore would argue that more systematic data might allow us to educate students more efficiently and perhaps even at lower cost in terms of staff and student time.

## Misc

R Benjamin Aldridge was supported by the Wellcome Trust (Reference 083928/Z/07/Z).

## Competing interests

The authors declare that they have no competing interests.

## Authors’ contributions

RBA was involved in the conception and design of the study, acquisition of data, interpretation of data, drafting of manuscript and approving final version. SSM was involved in acquisition of data and its analysis, reviewing manuscript and approving the final version. JLR was involved in the conception and design of the study, guidance on interpretation of data, revising the manuscript and approving the final version. All authors read and approved the final manuscript

## Pre-publication history

The pre-publication history for this paper can be accessed here:

http://www.biomedcentral.com/1472-6920/12/27/prepub
